# Can We Breed Cattle for Lower Bovine TB Infectivity?

**DOI:** 10.3389/fvets.2018.00310

**Published:** 2018-12-07

**Authors:** Smaragda Tsairidou, Adrian Allen, Georgios Banos, Mike Coffey, Osvaldo Anacleto, Andrew W. Byrne, Robin A. Skuce, Elizabeth J. Glass, John A. Woolliams, Andrea B. Doeschl-Wilson

**Affiliations:** ^1^The Roslin Institute and Royal (Dick) School of Veterinary Studies, University of Edinburgh, Edinburgh, United Kingdom; ^2^Agri-Food and Biosciences Institute, Belfast, United Kingdom; ^3^Scotland's Rural College, Midlothian, United Kingdom; ^4^Institute of Mathematical and Computer Sciences, University of São Paulo, São Paulo, Brazil

**Keywords:** disease resistance, disease control, animal breeding, infectivity, bovine Tuberculosis

## Abstract

Host resistance and infectivity are genetic traits affecting infectious disease transmission. This Perspective discusses the potential exploitation of genetic variation in cattle infectivity, in addition to resistance, to reduce the risk, and prevalence of bovine tuberculosis (bTB). In bTB, variability in *M. bovis* shedding has been previously reported in cattle and wildlife hosts (badgers and wild boars), but the observed differences were attributed to dose and route of infection, rather than host genetics. This article addresses the extent to which cattle infectivity may play a role in bTB transmission, and discusses the feasibility, and potential benefits from incorporating infectivity into breeding programmes. The underlying hypothesis is that bTB infectivity, like resistance, is partly controlled by genetics. Identifying and reducing the number of cattle with high genetic infectivity, could reduce further a major risk factor for herds exposed to bTB. We outline evidence in support of this hypothesis and describe methodologies for detecting and estimating genetic parameters for infectivity. Using genetic-epidemiological prediction models we discuss the potential benefits of selection for reduced infectivity and increased resistance in terms of practical field measures of epidemic risk and severity. Simulations predict that adding infectivity to the breeding programme could enhance and accelerate the reduction in breakdown risk compared to selection on resistance alone. Therefore, given the recent launch of genetic evaluations for bTB resistance and the UK government's goal to eradicate bTB, it is timely to consider the potential of integrating infectivity into breeding schemes.

## Introduction

Bovine tuberculosis (bTB) is a zoonotic disease, which can compromise both human health and international livestock trade. Zoonotic TB caused by *Mycobacterium bovis*, is responsible for an estimated 10–15% of human TB cases (1) and was estimated in 2016 as causing 12,500 deaths globally (2, 3). Addressing bTB infection in humans has been embedded within the United Nations Sustainable Development Goals 2016–2030 and World Health Organisation's (WHO) End TB Strategy framework, which employs a “One Health” approach aiming to end the global TB epidemic by 2030 (2–4).

In the UK, bTB has been the most pressing animal health problem, with financial losses amounting to over £175 m per annum (5). Tackling bTB has been a persistent challenge for the livestock industry, veterinary profession and policy-makers, and also the research community. The current national bTB eradication strategy involves the systematic testing of herds to identify and then remove infected cattle, and uses the Single Intradermal Comparative Cervical Test (SICCT), complemented by abattoir carcass inspections and, with increasing frequency, interferon-gamma testing. This surveillance regime has been successful in reducing disease spread in areas where bTB is prevalent and many EU countries and regions, including Scotland, have achieved Officially bTB Free (OTF) status (6). However, bTB persists in several regions (7) and herd incidence has increased in Wales, and also in High Risk and Edge areas in England (March 2018), despite the decrease in the overall herd incidence in England (8). Therefore, the continuing difficulties in eradicating bTB necessitate further exploration of additional disease control interventions that can complement existing strategies.

Selective breeding can complement classic disease control strategies, reducing the requirement for biosecurity measures and movement restrictions which have a major economic impact for herds undergoing a bTB breakdown (9, 10). Within the last few decades, breeding programmes (genetic and genomic selection) in livestock have achieved a remarkable improvement in production, e.g., milk yield in dairy cattle (11), and fitness traits such as fertility (12). Expanding the breeding objectives to include health and welfare traits offers new opportunities for disease control (10). The focus of genetic disease control so far has been on selection for improved resistance to becoming infected or diseased after exposure to pathogens. For example, by exploiting heritable genetic variation in disease resistance it has been possible to reduce mastitis incidence in cattle (13, 14) and mortality caused by infectious pancreatic necrosis in Atlantic salmon (15). Many studies have presented overwhelming evidence for genetic variation in resistance to bTB in cattle (16–19), which supports inclusion of bTB resistance in cattle breeding objectives in countries where bTB is prevalent. Recent efforts to combine national bTB surveillance and genetic data have enabled the publication of cattle evaluations for resistance to bTB in the UK (TB Advantage), which are currently used by farmers on a voluntary basis (20).

Veterinarians and epidemiologists have long considered reducing host infectiousness as an effective means to decrease disease transmission (21, 22). Infectiousness can be defined as the product of the contact rate between the infected individual and non-infected individuals, the propensity to transmit infection once infected (termed “infectivity” herein), and the duration of the infectious period (23, 24). For bTB, the contact rate between infected and non-infected herds is reduced by the movement restrictions imposed on herds with a bTB breakdown status. The duration of the infectious period is reduced by the test-and-cull policy which removes detectable infected animals, albeit with moderate animal-level sensitivity. In principle, infectivity can be reduced by vaccination, however, currently there are no vaccines (or subsequent tests) commercially available that allow differentiating between naturally infected and vaccinated individuals (i.e., a DIVA test) and would hence enable the safe use of vaccination for bTB control. Phenotypic variation in infectiousness is supported by numerous epidemiological studies showing that the Pareto principle commonly applies in epidemics, such that 20% of individuals are responsible for 80% of transmission events (22, 25–28). The individual differences in disease transmission are often attributed to different shedding patterns which may indicate phenotypic variation in host infectivity.

Emerging evidence suggests that infectivity can be, at least to some extent, under host genetic control (21, 29–33). Resistance and infectivity are thus two potentially distinct host genetic traits affecting disease transmission (see Table 1 for definitions and statistical and mechanistic distinctions between resistance and infectivity). Hence, if genetic variation in infectivity exists, can be estimated reliably, and has no significant impact on other desired traits, reduced infectivity could be a target for genetic improvement, in addition to disease resistance. Several authors have previously proposed (29, 34, 35) or demonstrated theoretically (36–39), that breeding livestock for both resistance and reduced infectivity can be an effective approach to reduce disease risk and prevalence.

**Table 1:** Mechanistic and statistical distinction between resistance and infectivity in the context of bTB.

	**Resistance**	**Infectivity**
Definition (generic)	Propensity of an individual to become infected, given exposure	Propensity of an individual, once infected, to transmit infection to non-infected group members
Interpretation (bTB context)	For a given uniform level of exposure, a more resistant cow has lower risk of becoming *M. bovis* infected than a cow with low resistance	Given uniform contact rates and duration of infectious period, group members exposed to an infected cow with high infectivity have a greater risk of becoming *M. bovis* infected than when exposed to an infected cow with low infectivity
Disease phenotypes used in statistical models to infer trait estimates	Individuals' bTB infection status, based on ante-mortem test results, measured at multiple time points throughout a breakdown, possibly combined with post-mortem test results	
Trait contribution to disease phenotype	Only affects a cow's own infection status (direct effect on own disease phenotype)	Can only affect the infection status of group members (indirect effect on disease phenotype of group member)
Underlying mechanisms	Unknown; Speculated to be related to mechanisms affecting bacterial entry, establishment and within-host replication	Unknown; Speculated to be related to mechanisms controlling bacterial shedding patterns

In this Perspectives article, we (a) review existing evidence that cattle may genetically differ in their bTB infectivity, (b) outline data and methodology requirements for estimating genetic infectivity for bTB, (c) discuss the benefits from considering infectivity in genetic evaluations, and (d) identify key challenges and future research opportunities for incorporating infectivity, in addition to resistance, in cattle breeding programmes aiming to reduce bTB prevalence.

### Emerging Evidence That Infectivity Is Genetically Controlled

In bTB, differences in shedding patterns of *M. bovis* have been reported in various studies, but those have been mostly attributed to phenotypic variation rather than host genetics. For example, the number and frequency of episodes of shedding of *M. bovis* in cattle, were found to be dose- and infection route-dependent (40). Even amongst controlled experimentally infected calves, significant variation in shedding patterns have been described amongst individuals when presented with the same dose and infection route (41). In wild boars, the intensity and shedding of mycobacteria from the *M. tuberculosis* complex were found to affect the probability of new infections, while shedding intensity was shedding-route-dependant (42). In badgers, heterogeneity in shedding was found between different social groups (43) which may indicate family, and hence genetic, differences in shedding. Other studies found that the type of tuberculous lesions developed can affect the potential of infected individuals for transmitting infection (44), while evidence suggests that cattle with and without confirmed lesions may constitute, at least to some extent, genetically different subpopulations (45). Heterogeneity in lesion formation and stability of infected individuals suggests variation in mechanisms underlying infectivity rather than resistance, as less stable lesions are more prone to breaking open and thus to higher bacterial shedding. Human tuberculosis epidemiology is consistent with the existence of *M. tuberculosis* super-spreaders (46, 47), which may indicate the existence of individuals with high infectivity. In bTB epidemiological studies, the best model fit has been observed when accounting for *M. bovis* super-spreaders (48, 49), and super-spreading has been proposed for badgers and other wildlife species (7, 50, 51). However, there remains a controversy about the existence of super-spreaders in bTB (52).

In other diseases, genetic variation in infectivity was found to manifest itself in various ways, such as through genetic differences in the potential for, quantity and type of infectious material shed by infected hosts. For example, genetic variation was found in the fecal egg count of sheep artificially infected with the same gastro-intestinal parasite strain and dose (53, 54). Furthermore, in cases of hosts infected with more than one genotype of the same pathogen, host immune response can affect pathogen strain competition and diversity with subsequent effect on host infectivity (55). More direct evidence for genetic differences in host infectivity has been recently obtained from transmission experiments of viral and protozoal infections in fish (31, 33). In these studies fish were found to differ in their probability of becoming diseased depending on the family or genotype of the initially infected fish that seeded the infection.

In summary, phenotypic variation in host infectivity is a common phenomenon, and for some diseases, this was shown to encompass genetic variation. In bTB, phenotypic variation in *M. bovis* shedding has been demonstrated by a few studies, but the extent to which this variation is due to cattle genetics is currently unknown. It is possible that host disease resistance and infectivity share some common genetic pathways controlling pathogen replication and consequently shedding (Table 1). This raises the question as to what extent bTB infectivity and resistance are genetically correlated, and how combined resistance and infectivity can affect bTB transmission.

### Data and Methodology Requirements for Estimating Genetic Effects for bTB Infectivity

Infectivity, referring to an individual's ability to transmit infection (Table 1), is difficult to measure directly from field data where transmission routes (who-infects-whom) are difficult to trace and many transmissions are not observed or detected. Infectivity phenotypes can be obtained by measuring individual shedding rates (56). Measuring shedding has only been practical in special cases, e.g., fecal egg count for nematodes, and is very challenging if carried out routinely on the scale of sample sizes needed to inform breeding programmes.

However, shedding is not the only phenotype that can be used to track infectivity. Instead, it is possible to estimate genetic variation in infectivity by monitoring the progression of infection, i.e., the infection status of individuals, in different herds over time. Recently, novel inference methods have been developed to simultaneously estimate and untangle genetic effects for resistance and infectivity from longitudinal data of individual infection status (Table 1) (34, 36, 57, 58).

Common requirements for estimating genetic variation in infectivity using these novel methods are that (i) genetically related individuals are spread over different epidemics (herds/breakdowns), (ii) individual epidemics occur in “closed” groups with minimum between-group transmission, and (iii) individual infection times differ, and are known or can be inferred. These requirements appear to be satisfied by bTB. The UK national bTB eradication scheme has generated systematic repeated records of SICCT test results for a large number of herds containing related animals. In addition, due to movement restrictions imposed on herds undergoing a breakdown, herds can be considered as closed groups during the breakdown period, and data collected can be used to infer infectivity. Although the exact time of cattle infection with *M. bovis* is unknown, the repeated SICCT testing during this period provides longitudinal measurements of individuals' infection status, from which infection times can be inferred using Bayesian inference and data augmentation methods (34, 58).

It remains to be tested with field bTB data, how various sources of uncertainty affect genetic infectivity estimates. For example, these methods assume knowledge of the true infection status of an individual, which raises the question whether SICCT and other monitoring records are appropriate for this purpose. Of these, SICCT is the most commonly available measurement but its relatively poor sensitivity is well documented, i.e., its ability to correctly identify infected individuals; published sensitivity estimates range from 26 to 91% (59–63). Whether the test result reflects the true infection status of an animal is under on-going investigation within the bTB research community. Nevertheless, the positive predictive value of the test, i.e., the proportion of individuals that test positively and truly have the disease, is sufficiently high that false positives are likely to be few amongst the observed reactors. The specificity of SICCT in officially tuberculosis free herds has been estimated to be 99.98% (64). Therefore, already recorded SICCT phenotypes can provide information to search for genetic effects associated with infectivity. Including information from culling associated with SICCT testing has proven adequate for obtaining sufficiently accurate estimated breeding values (EBVs) for bTB resistance in the current bTB genetic evaluations (20). Expanding these evaluations to consider both resistance and infectivity would be expected to be beneficial primarily in high bTB risk areas, where the positive predictive value of SICCT is higher due to the higher disease prevalence (65).

### Expected Benefits From Implementing Infectivity as an Additional Disease Phenotype in Genetic Evaluations for bTB Control

bTB has been a seemingly intractable problem in the UK in recent decades and understanding how cattle genetics influences bTB spread is important for eradication. Under the hypothesis that some cattle infected with bTB are genetically more infectious than others, reducing the occurrence of cattle with higher genetic infectivity through selective breeding would (i) reduce bTB transmission between cattle by removing highly infectious individuals comprising a major risk factor for herds, (ii) reduce shedding of *M. bovis* and hence reduce a major source of infection for the environment (30) and wildlife vectors, e.g., indirectly reduce bTB spill over to badgers. Badgers are susceptible to *M. bovis* infection and reducing infectivity in cattle should also reduce the pathogen burden in the environment (shedding e.g., in milk, feces, air, etc.).

If we were to estimate infectivity effects, it would enable breeders and farmers to select bulls whose offspring are not only expected to be less likely to become infected (more resistant), but also less likely to transmit bTB infection, if infected. Selection on breeding values for resistance and infectivity is expected to reduce the population R_0_ (37, 39), i.e., the expected number of secondary cases produced by a typical infectious individual in a completely susceptible population (66), hence contributing to disease control. However, bTB transmission occurs within and across different species. Hence the overall bTB *R*_0_ is composed of *R*_0_*cattle*__, *R*_0_*badgers*__, *R*_0_*cattle*−*to*−*badgers*__, and *R*_0_*badgers*−*to*−*cattle*__ (67, 68), where the cross-species relationships warrant further investigation. Reducing cattle infectivity would be expected to reduce *R*_0_*cattle*__ and *R*_0_*cattle*−*to*−*badgers*__, as well as the infection feedback loop from *R*_0_*badgers*−*to*−*cattle*__. A small reduction in each component may suffice to bring the overall *R*_0_ to below 1 and make the risk of new breakdowns negligible (10, 69). Investigating individual differences in infectivity might shed light on the variation observed in herd bTB prevalence and the relationship of infectivity with detectable bTB status, and why in some herds bTB persists with recurrent/chronic breakdowns, while other herds appear to be able to rapidly clear infection. Investigating variation in infectivity would also shed light on the weather bTB super-spreaders exist, as animals at the tail of the distribution would be “super spreaders” relative to all others, and what is their role in bTB spread.

**Figure 1:**
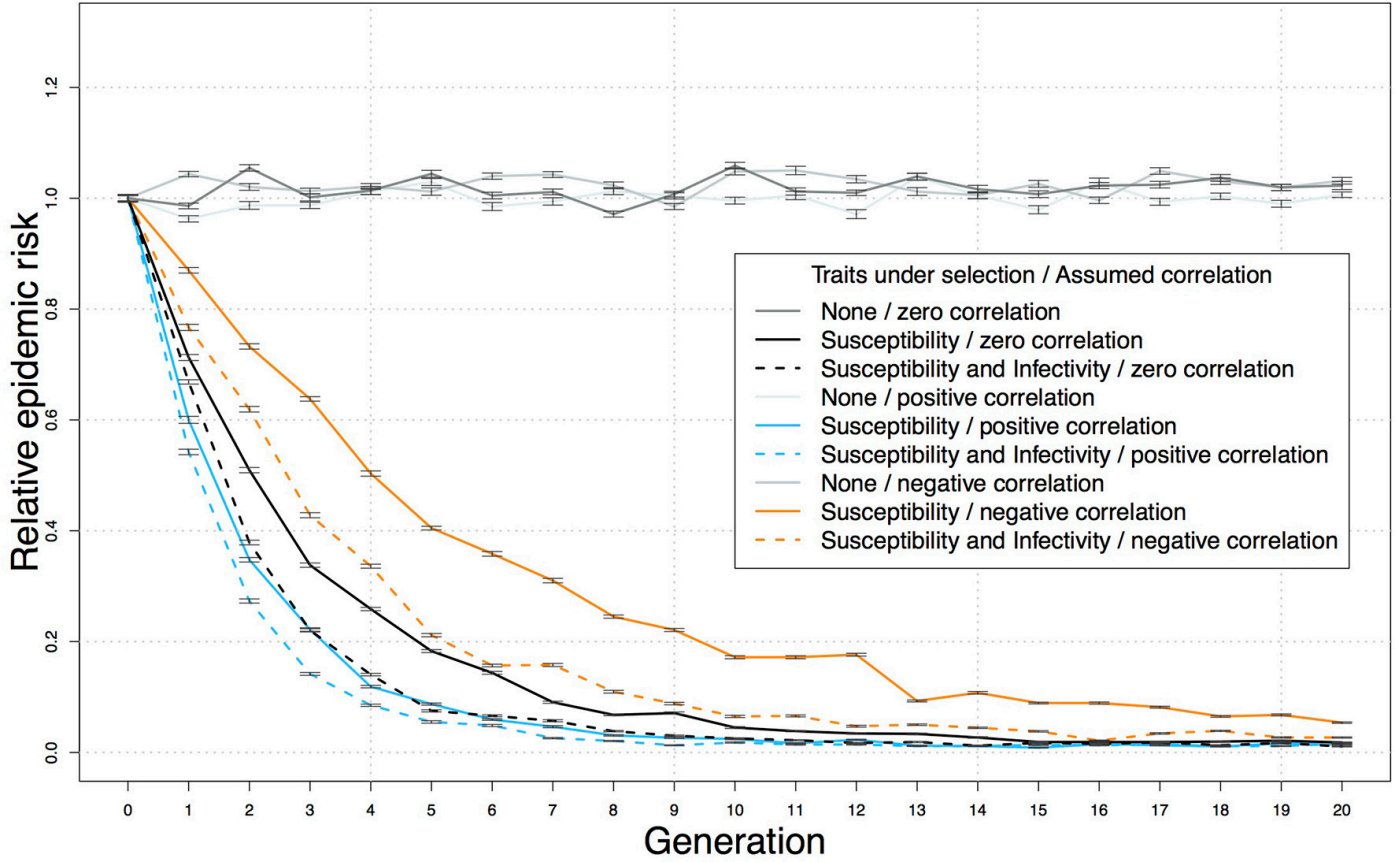
Reduction in the predicted relative risk of bTB breakdown in a herd, following introduction of an infected cow, over 20 generations of selection for resistance and lower infectivity, or for resistance alone. Predictions from a stochastic genetic-epidemiological simulation model incorporating genetic variation in resistance and infectivity (38), comprising populations of 10,000 half-sib individuals randomly distributed into 100 herds of the same size. Means and standard errors were obtained over 50 replicates. The parameter values were based on the British genetic evaluations for bTB resistance (20) and a previous genetic-epidemiological model (70) as follows: bTB testing intervals of 60 days, SICCT sensitivity of 60%, 50% selection on the sires, accuracy of 0.5 and latent heritability of 0.6 [corresponding to heritability for the observed indicator traits of below 0.2 (70)] for both resistance and infectivity, economic values of one for both traits, and external force of infection of 5 × 10^−5^ (70). Each breakdown was initiated by one infectious individual, and variance and accuracy were assumed constant over generations. The correlation between resistance and infectivity was assumed to be zero, 0.5 or −0.5.

A genetic-epidemiological simulation model can be used to assess the relative benefits of using a selection index that includes both resistance and infectivity compared to selecting on resistance alone. For this purpose we extended a stochastic epidemiological Susceptible-Latent-Infectious-Test sensitive model for bTB that originally assumed genetic variation only in resistance (70) with parameter values from the British genetic evaluations for bTB resistance (20) to incorporate hypothetical additional variation in infectivity (34, 38). We then used this model to simulate bTB spread in each herd and predict the impact of selection on breakdown risk, defined by the proportion of simulated bTB epidemics where infected index cases generated secondary cases. This is pertinent as field characteristics of epidemics often show curvilinear responses to control strategies. We found that when adding infectivity alongside resistance to the breeding objective, the reduction of the risk of a bTB breakdown was substantial and more pronounced in the early generations (Figure 1) (34, 38). For example, assuming 50% selection on sires, moderate heritabilities and prediction accuracies for resistance and infectivity, and zero correlation between resistance and infectivity, the relative epidemic risk at generation 5 was ~0.2 with selection for resistance alone, but < 0.1 for combined selection for both resistance and infectivity, even when external sources of infection were included (Figure 1). These simulations designed as proof-of-principle, provide a crude estimate of the predicted effects which will depend on e.g., the magnitude of the genetic variance in the objective traits and various demographic factors. However, these findings are indicative that by targeting both resistance and infectivity in combination, disease control benefits can be of larger magnitude (i.e., more effective) and more responsive (i.e., quicker to see results) (38).

The epidemiological benefits and additional gain expected from adding infectivity to the breeding goal depends on its genetic correlation with other traits of economic interest. A classic example of the impact of adverse correlated responses, is the reduction of cattle fertility following selection on milk yield, due to adverse genetic correlation with milk yield (12). Based on estimated genetic correlations among traits, genetic selection for enhanced bTB resistance is not expected to adversely affect other traits in the breeding goal (16, 20), and was found to be unlikely to change the probability of correctly identifying non-infected animals via the SICCT diagnostic test (71). However, genetic correlations between resistance and infectivity may affect the outcome of genetic bTB control. Indeed, based on the genetic-epidemiological bTB model described above (38) the strongest benefit of adding infectivity into the selection criterion compared to selecting on resistance alone is observed in the case of an adverse genetic correlation between the traits (Figure 1), and this is because the progress achieved by breeding schemes targeting only resistance would be delayed due to an indirect increase in infectivity. Considering breeding values for infectivity can help alleviate this delay and accelerate progress toward disease eradication (38).

### Future Opportunities and Challenges

In principle, bTB surveillance schemes such as those implemented in the UK, RoI, and NZ, would permit researchers to pioneer the estimation of infectivity genetic effects without the need to collect new data. Current genetic evaluations for bTB resistance (20) use phenotype and pedigree information, with increasing amounts of genomic data. As the incidence of bTB reduces, the information obtained from pedigrees will also reduce. Therefore, genomic information becomes increasingly vital, and high-density genomic data can now be obtained cost-effectively by genotype imputation (72, 73). It has been shown that genomic prediction for bTB resistance using genomic information is feasible (18), and prediction accuracies can be improved by using larger training sets of genotyped animals and genome sequence information. This is pertinent for infectivity, as it has been shown in simulation studies that the prediction accuracy for infectivity is expected, at least initially, to be modest (34). This genomic information also allows investigation of the genetic architecture of bTB infectivity and the search for causal variants.

Challenges arising in the analysis of bTB data to uncover genetic infectivity include accounting for multiple and poorly understood transmission routes of *M. bovis*, and obtaining more reliable disease phenotypes. Separating the effects of the infectious dose from host response is extremely challenging in field situations where exposure may not be uniform (74). However, sophisticated Bayesian inference methods, coupled with phylodynamics and *M. bovis* genome sequence information, can help to infer transmission routes and obtain information on the networks of who-infects-whom (49, 75–78), which is useful for predicting infectivity (79). More reliable disease phenotypes could be obtained by quality control on individual tester performance to improve the consistency of data recording on the farms (80), and by developing improved diagnostics. Machine learning techniques (Deep Learning) hold the promise that sufficiently accurate disease phenotypes can be obtained in a cost-effective manner for large sample sizes using routinely collected mid infra-red spectral data from milk recording (Coffey M. personal communication October 2018). Together, continuous development of improved diagnostic and modeling tools provide promising prospects for genetic bTB control.

## Conclusion

Host infectivity is an important trait for disease transmission and emerging evidence suggests that it may be under genetic control to some extent; however, the role of genetic infectivity of cattle in bTB spread remains to be explored. Infectivity might be difficult to capture from noisy field data; however, the UK bTB surveillance database and newly developed statistical methods provide the opportunity to estimate genetic effects for infectivity. Exploiting genetic variation in infectivity as a complementary bTB control method is a low-investment high-return approach, as it can be developed at minimal cost using data already available. Simulation studies suggest that breeding for both disease resistance and infectivity can complement and substantially enhance current disease control approaches toward bTB eradication. Using UK data to determine genetic regulation of disease transmission can create a platform for controlling bTB in other countries and for controlling other infectious diseases.

## Author Contributions

ST, AA, RS, GB, JW, and AD-W conceived the perspectives. ST drafted the manuscript, designed and carried out the simulations and participated in the interpretation of findings. JW and AD-W contributed to the initial draft and simulation designs. All authors contributed to later versions of the manuscript, read and approved the final manuscript.

### Conflict of Interest Statement

The authors declare that the research was conducted in the absence of any commercial or financial relationships that could be construed as a potential conflict of interest.
